# Aging and antioxidants: the impact of dietary carotenoid intakes on soluble klotho levels in aged adults

**DOI:** 10.3389/fendo.2023.1283722

**Published:** 2023-10-26

**Authors:** Xingkang He, Xin Yin, Xin Chen, Xiaoli Chen

**Affiliations:** ^1^ Department of Gastroenterology, Sir Run Run Shaw Hospital, Zhejiang University Medical School, Hangzhou, China; ^2^ Department of Radiation Oncology, the First Affiliated Hospital, School of Medicine, Zhejiang University, Hangzhou, Zhejiang, China

**Keywords:** carotenoids, antioxidants, klotho, NHANES, aging

## Abstract

**Objectives:**

The association between dietary carotenoid intake and Soluble Klotho (S-Klotho) levels among the elderly population requires further evaluation. The purpose of this study is to evaluate the relationship between the dietary carotenoid intake and the S-Klotho plasma levels in older adults.

**Methods:**

Eligible participants aged 60 years or above were selected from the National Health and Nutrition Examination Surveys (NHANES) data, collected between 2007 and 2016. The consumption of carotenoids was determined through two 24-hour dietary recall assessments. Moreover, the S-Klotho levels in the serum were measured using an Enzyme-Linked Immuno-Sorbent Assay (ELISA).

**Results:**

A total of 5,056 participants were included in the study having a median total carotenoid intake of 9775.25 μg (95% confidence interval (CI): 8971.30−10579.21) and a median S-Klotho concentration of 815.59 pg/mL (95% CI: 802.59−828.60). The multivariable regression analysis showed that a single standard deviation increase in total carotenoid intake was significantly associated with an 8.40 pg/mL increase in S-Klotho levels (95% CI: 0.48−16.31). When the carotenoids were divided into quartiles, participants in the third ((4963.5μg/day,11662.5μg/day]) and fourth quartiles ((11662.5μg/day,377178μg/day]) showed higher S-Klotho levels compared to those in the first quartile. Among carotenoid subtypes, increased intake of α-carotene, β-carotene, and lutein with zeaxanthin was associated with elevated S-Klotho levels. These observed associations between carotenoid subtypes and S-Klotho levels remained consistent across male participants, having a normal weight, and a moderate physical activity based on stratified analysis.

**Conclusion:**

The total carotenoid intake was positively related to plasma levels of S-Klotho in the elderly population, particularly for α-carotene, β-carotene, and lutein with zeaxanthin. However, further research is needed to confirm these findings and explore the underlying mechanisms behind this relationship.

## Introduction

Aging is a multifactorial process that is characterized by its complexity. One of the proposed mechanisms underlying this process is the changes in oxidative stress and inflammation that occur with advancing age (1). Klotho is a type-I membrane protein that is linked to β-glucuronidases and is produced by the klotho gene (2). This protein is a regulator for aging, discovered by Kuro-o and his colleagues in a mouse model with genetic defects, resulting in several premature aging syndromes and shortened lifespans (3). Moreover, Soluble Klotho (S-Klotho) is produced through the cleavage of the membrane-bound Klotho and can be detected in the blood, cerebrospinal fluid, and urine (4). It is also involved in the regulation of oxidative stress, inflammation, and aging, and a low level of S-Klotho is associated with various metabolic and aging-related diseases, such as type 2 diabetes, Non-Alcoholic Fatty Liver Disease, cardiovascular disease, and cognitive regression (5–10). Furthermore, a low level of S-Klotho yields in an increase in the risk of all-cause and, most importantly, the cardiovascular disease-related mortality (11–13). The levels of systemic inflammation and S-Klotho in the plasma are inversely correlated; the decrease of the S-Klotho levels in the plasma is associated with systemic inflammation increase and vice versa (14).

In opposite, carotenoids are organic pigmented molecules that arise from fungi, bacteria, and plastids of algae and plants (15). They are well-known for their antioxidant properties and their ability to protect the human being against oxidative stress and inflammation (16). These molecules play a crucial role in sustaining human health and increasing the lifespan of animal models (17–19). Since the human body cannot synthesize carotenoids, these latter must be acquired from dietary sources (20) where, among the most well-known carotenoids, one can find the β-carotene, lycopene, lutein, and zeaxanthin. Finally, due to their antioxidant properties, it is believed that carotenoids can assist in reducing the severity of chronic illnesses (21).

Despite the numerous benefits that carotenoids offer in terms of combating oxidative stress, inflammation, and their general positive influence on human health (17), the connection between the consumption of dietary carotenoids and S-Klotho plasma levels in aged adults remains largely unexplored. Therefore, the purpose of this study is to explore the link between carotenoid intake in the diet and the S-Klotho plasma levels among aged adults. The obtained results could provide valuable information regarding the potential impact of carotenoid-rich diets on aging and aging-related diseases. Furthermore, understanding the associations between dietary carotenoid intakes and S-Klotho levels in aged adults could have important implications for the development of dietary interventions yielding to promote healthy aging and prevent aging-related diseases.

## Methodology

### Study population

The National Health and Nutrition Examination Survey (NHANES) is a comprehensive study that evaluates the health and nutritional conditions of the non-institutionalized population in the USA. The survey uses a multi-stage and stratified probability design to obtain a representative sample of the US population. The protocols and the investigation plan used in the NHANES have been approved by the National Center for Health Statistics Ethics Review Board (https://www.cdc.gov/nchs/nhanes/irba98.htm) where all participants have to provide their informed consent in the survey. The data used in this study was obtained from five independent cross-sectional waves of NHANES (2007-2008, 2009-2010, 2011-2012, 2013-2014, and 2015-2016) and it was freely available. Participants over the age of 60 years were included in the study, and those who declined consent to participate or who had missing data on carotenoid intake, serum S-Klotho level, demographic information, or health status were excluded.

### Measurement of the dietary total carotenoid and subgenus intake

In the NHANES study, the dietary information of the participants was obtained through two 24-hour recall interviews. The protocol and data collection methods for the interviews are outlined in the NHANES dietary interviewers’ procedure manual, which can be found at this link: https://www.cdc.gov/nchs/nhanes/measuring_guides_dri/measuringguides.htm.The initial dietary recall interview was conducted face-to-face at the Mobile Examination Center. During this interview, participants were asked to provide a detailed description of all the food and beverages consumed within the preceding 24 hours, including the type and quantity of each item. Following the first interview, a second interview was conducted via telephone after a gap of three to ten days. During this second interview, participants were asked to provide a comprehensive breakdown of the specific types and amounts of each food they had consumed over the past 24 hours. The accuracy and reliability of the technique applied in this study have been ascertained by other investigations that also used NHANES data (22–24). Intakes of carotenoids and other nutrient data were derived from the US Department of Agriculture Food and Nutrient Database for Dietary Studies of NHANES (25). The total carotenoids considered in the study included α-carotene, lycopene, β-cryptoxanthin, β-carotene, and lutein with zeaxanthin. To calculate the intake of these compounds, the results from the two 24-hour recall periods were averaged. For each NHANES cycle, dietary intakes of lutein, zeaxanthin, and lycopene from supplements were documented during two 24-hour recall periods (26). In this study, the average intake of lycopene, lutein, and zeaxanthin supplements was taken over two days, and the total intake of these compounds was determined by summing both dietary and supplemental intakes. Finally, the total dietary carotenoid intake was determined by summing up the intake of α-carotene, lycopene, lutein, and zeaxanthin, β-carotene, and β-cryptoxanthin from food sources, while the total carotenoid intake was ascertained by summing up the carotenoid intake from both dietary sources and supplements. The selection of two 24-hour recall interviews was based on the need to obtain more precise and representative dietary information from participants. Having interviews on different days enables to take into account the variations in dietary intake within an individual and offers a more comprehensive evaluation of the typical dietary habits of the participants.

### Determination of S-Klotho concentrations

Specimens were collected from the antecubital vein of the participants who were in a horizontal position. Before the sample was taken, participants were instructed to fast for 12 hours, avoid drugs and caffeine, to have a specific dinner, and to refrain from moderate physical activity within 24 hours and vigorous activity within 48 hours. The S-Klotho levels were ascertained by employing a solid-phase sandwich Enzyme-Linked Immuno-Sorbent Assay (ELISA) kit from Demeditec (Kiel, Germany), as per the manufacturer’s instructions (https://wwwn.cdc.gov/Nchs/Nhanes/2015-2016/SSKL_I.htm). To evaluate the accuracy of the test, the intra- and inter-assay coefficients of variation were calculated to get two doses of purified S-Klotho.

### Assessment of covariates

The following important covariates were included for adjustment in this study: the year of the NHANES cycle, age, gender (male and female), ethnicity (non-Hispanic white, Mexican American, non-Hispanic black, and other ethnicities), marital status (unmarried and married), educational level (grade or less, high school, some college, and college or more), family income-to-poverty ratio, Body Mass Index (BMI), smoking status (never, former, and now smoking), drinking status (never, former, mild, moderate, and heavy drinking), energy intake, estimated Glomerular Filtration Rate (eGFR), physical activity, use of medication, systemic immune-inflammation index (SII), serum 25-hydroxyvitamin D, and self-reported chronic diseases including hypertension, diabetes, stroke, heart attack, congestive heart failure, coronary heart disease, and cancer. Unmarried individuals regroup those who were never married, divorced, widowed, living with a partner, and separated. As for the physical activity, it was categorized as either with or without moderate physical activity. Finally, the BMI variable was divided into four categories in the stratified analysis: underweight (<18.5 kg/m2), normal weight (18.5 to <25 kg/m2), overweight (25 to <30 kg/m2), and Obesity (≥30 kg/m2).The systemic immune-inflammation index (SII) is a new indicator of systemic inflammation, calculated by multiplying the platelet count and neutrophil count and dividing the result by the lymphocyte count (27). This index offered a more comprehensive and balanced representation of a person’s immunological and inflammatory responses (28).

### Statistical analysis

Using sample weights, strata, and primary sampling units, the analysis was conducted to ensure the accurate national estimates. The sample characteristics were displayed as mean values with 95% Confidence Intervals (CIs) for continuous variables and as percentages for categorical variables. Carotenoids were analyzed both as a continuous variable and as a categorical variable split into four quartiles, with the lowest quartile considered as the basis for comparison. Quartiles of carotenoid intakes (including the total carotenoid and its subgroups) were established according to the distribution among the study population. We have taken into consideration whether adjustments were necessary for multiple testing, if applicable. Multivariable linear regression models were employed to evaluate the association between carotenoids and the plasma level of S-Klotho. Moreover, three statistical models were constructed: a crude model with no adjustments (Model I), an adjusted model that takes into account the year and the age of the participant (Model II), and a further adjusted model that incorporated the year, age, gender, ethnicity, marital status, education level, family income-to-poverty ratio, smoking status, drinking status, eGFR, BMI, total energy intake, physical activity, medication use, SII, serum 25-hydroxyvitamin D, and self-reported chronic diseases (Model III). In addition to total carotenoids, the effects of the carotenoid subgroups on the S-Klotho level were also assessed. To examine if the association between the carotenoid subgenera and S-Klotho level was modified by some variables, such as sex, BMI, and physical activity, stratified analyses sorted by sex (male or female), BMI (normal weight, overweight, obesity), and physical activity (with or without moderate physical activity) were also conducted. Finally, to evaluate the robustness of our findings, we executed a sensitivity analysis that incorporated several macronutrients (carbohydrates, proteins, fat, and sugars) into our multivariable linear regression models. All analyses were performed using R (version 4.20) software. A two-tailed P value lower than 0.05 was considered to be a statistically significant result.

## Results

### Baseline characteristics of included participants

**Figure 1:**
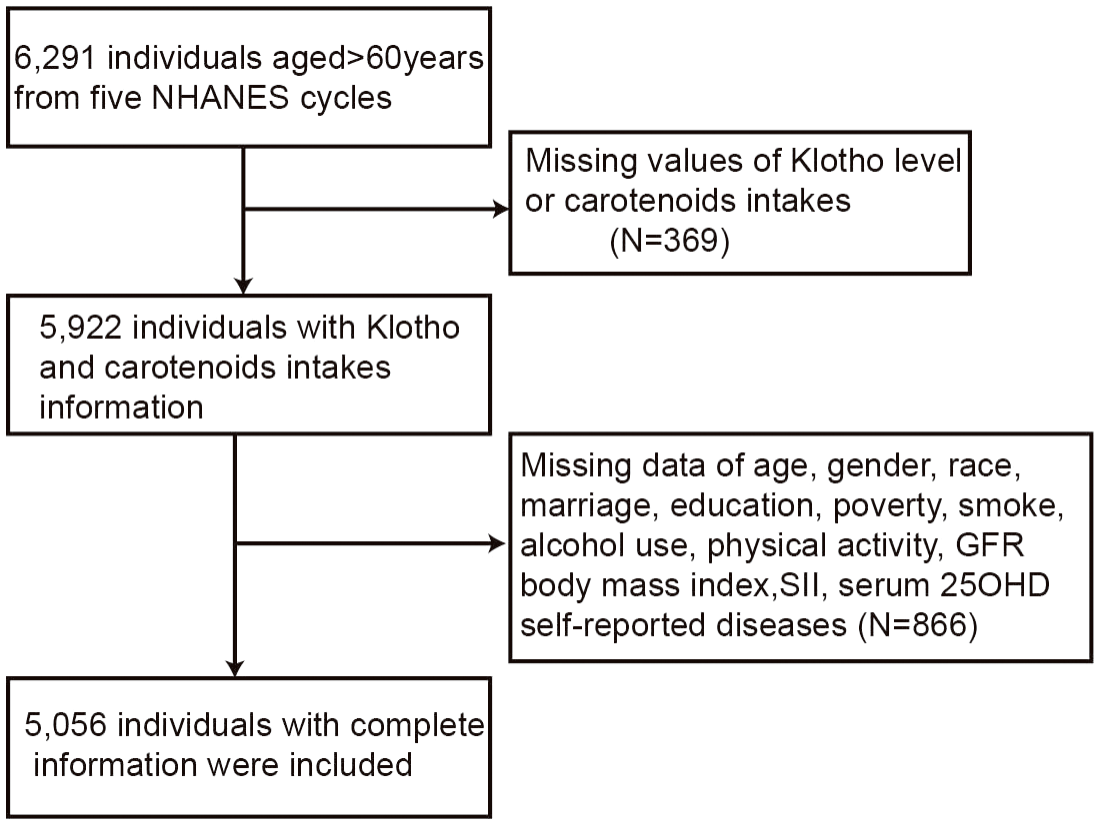
Flowchart of included participant selection.

A total of 5,056 elderly individuals were recruited for this study, and the selection of the study cohort has been demonstrated in **Figure 1**. **Table 1** shows the detailed characteristics of the included elderly population based on quartiles of dietary carotenoid intake. The average age of participants was 67.5 years, and their average annual family income was more than three times the poverty level (mean income/poverty ratio = 3.20). The daily energy intake was recorded to be an average of 1899.36 kcal, with a mean eGFR of 75.94 mL/min/1.73m^2^, BMI of 29.62 kg/m^2^, total carotenoid intake of 9775.25 μg per day, and S-Klotho concentration of 815.59 pg/ml. Individuals who consume a greater amount of carotenoids in their diet were more likely to exhibit certain demographic and lifestyle characteristics. Specifically, individuals with high intake of carotenoids tended to be younger, female, non-Hispanic White, and possess a higher socioeconomic status (including higher educational levels, marriage, and income). Additionally, those with high intake of carotenoids had higher serum 25-hydroxyvitamin D levels, lower SII, and were less likely to smoke, while engaging in moderate drinking, physical activity, and medication use, and consuming more energy overall. Furthermore, participants with a large consumption of carotenoids displayed a higher serum Klotho level than those who consumed a smaller amount, although the difference was not statistically significant. **Table S1** provided a summary of the characteristics exhibited by the elderly participants who were included in the study, based on the quartiles of S-Klotho concentration. In terms of carotenoid subgroups, the average daily consumption of α-carotene, β-carotene, β-cryptoxanthin, lycopene, and lutein with zeaxanthin was 477.3, 2,548.41, 88.4, 4,943.17, and 1,717.97 micrograms, respectively.

**Table 1:** Characteristics of included population based on quartile of dietary carotenoid intake in the NHANES (N =5,056).

		Quartile of dietary carotenoid intake (μg/day)				
Characteristic	Overall, N = 5056 (100%)	Q1 N = 1265 (25%)	Q2 N = 1263 (25 %)	Q3 N = 1264(25%)	Q4 N = 1264 (25%)	P Value
Age (years)	67.50(67.27,67.73)	67.93(67.45,68.41)	67.25(66.89,67.62)	67.69(67.30,68.08)	67.20(66.79,67.60)	0.04
Gender %						<0.01
Female	52.83	54.36	56.01	53.73	47.79	
Male	47.17	45.64	43.99	46.27	52.21	
Ethnicity %						<0.01
Non-Hispanic White	80.35	75.53	80.31	82.32	82.29	
Mexican American	4.11	4.48	3.72	4.68	3.62	
Non-Hispanic Black	7.53	11.28	7.3	5.87	6.38	
Other ethnicities	8.02	8.71	8.67	7.12	7.71	
Education %						< 0.01
Grade or less	16.57	22.86	16.83	15.36	12.53	
High school	22.94	28.42	22.41	21.62	20.39	
Some college	31	31.26	33.01	32.92	27.06	
College or more	29.5	17.46	27.75	30.1	40.02	
Married status %	66.73	60.37	65.27	68.95	70.98	<0.01
Poverty income ratio	3.20(3.09,3.30)	2.67(2.52,2.82)	3.19(3.06,3.32)	3.34(3.18,3.49)	3.49(3.34,3.64)	< 0.01
Smoking status %						< 0.01
Never	12.66	14.49	14.28	11.7	10.63	
Former	40.61	36.36	39.68	43.05	42.49	
Now	12.34	20.02	11.5	10.69	8.65	
Drinking status %						< 0.01
Never	12.66	14.49	14.28	11.7	10.63	
Former	22.25	28	24.38	19.83	18.03	
Mild	45.54	36	45.02	47.42	51.73	
Moderate	12.71	12.96	11.29	13.68	12.92	
Heavy	6.84	8.55	5.03	7.37	6.69	
Body Mass Index (kg/m2)	29.62(29.31,29.93)	29.85(29.35,30.36)	29.84(29.22,30.46)	29.80(29.33,30.26)	29.07(28.57,29.56)	0.08
Energy intake (kcal/day)	1899.36 (1867.60,1931.12)	1670.82 (1612.52,1729.12)	1836.50 (1769.30,1903.71)	1907.94 (1859.43,1956.45)	2129.89 (2069.76,2190.02)	< 0.01
Physical activity (Yes) %	70.66	63.59	72.36	72.77	72.64	<0.01
Glomerular filtration rate (ml/min/1.73m^2^)	75.94 (75.12,76.77)	74.81 (73.36,76.25)	75.96 (74.71,77.21)	75.86 (74.30,77.42)	76.90 (75.69,78.11)	0.16
Serum 25-hydroxyvitamin D (nmol/L)	78.31 (76.58,80.05)	74.02 (71.47,76.58)	77.76 (75.04,80.48)	81.04 (78.88,83.21)	79.60 (76.87,82.34)	< 0.01
Systemic immune-inflammation index	553.67 (539.27,568.08)	581.23 (546.18,616.29)	566.62 (535.64,597.61)	551.73 (527.65,575.80)	521.71 (501.85,541.57)	0.01
Use of medication %	85.67	85.33	85.54	87.59	84.23	0.41
S-Klotho level (pg/ml)	815.59 (802.59,828.60)	790.82 (773.13,808.51)	819.91 (797.43,842.39)	814.93 (794.51,835.34)	831.67 (806.78,856.56)	0.06
Total Carotenoid (μg/day)	9775.25 (8971.30,10579.21)	823.71 (790.56, 856.86)	3227.55 (3162.43, 3292.68)	7711.46 (7576.70, 7846.22)	24929.65 (22767.87,27091.44)	< 0.01
α-Carotene (μg/day)	477.30 (389.28,565.32)	33.87 (28.19, 39.55)	159.88 (141.04, 178.73)	526.14 (481.39, 570.89)	1077.25 (783.97,1370.54)	< 0.01
β-Carotene (μg/day)	2548.41 (2255.66,2841.16)	225.47 (210.47, 240.46)	847.46 (789.32, 905.60)	2329.24 (2207.83,2450.64)	6180.60 (5251.01,7110.19)	< 0.01
β-Cryptoxanthin (μg/day)	88.40 (80.42,96.39)	36.67 (31.14, 42.21)	76.61 ( 66.35, 86.86)	98.34 (81.90,114.79)	130.67 (108.21,153.12)	< 0.01
Lycopene (μg/day)	4943.17 (4524.48,5361.86)	136.74 (112.71, 160.78)	1293.31 (1198.89, 1387.74)	3378.04 (3194.07, 3562.01)	13642.37 (12444.97,14839.76)	< 0.01
Lutein with zeaxanthin (μg/day)	1717.97 (1536.96,1898.97)	390.96 (362.57, 419.34)	850.29 (787.99, 912.59)	1379.69 (1281.66,1477.73)	3898.76 (3350.39,4447.14)	< 0.01
Self-reported chronic diseases						
CHD %	8.83	9.47	7.81	8.99	9.13	0.73
Cancer %	23.64	24.13	22.95	24.46	23.11	0.89
CHF %	5.67	6.26	6.17	6.19	4.25	0.20
Stroke %	5.87	6.69	7.06	4.1	5.79	0.04
Heart attack %	7.34	8.26	7.1	7.51	6.68	0.72
Hypertension %	56.73	59.5	56.12	56.84	55.03	0.51
Diabetes mellitus %	19.43	18.91	20.29	19.85	18.64	0.34

Continuous variables are described as means ± 95%CI, and categorical variables are presented as percentages. All estimates accounted for complex survey designs. CHF, Congestive heart failure; CHD, Coronary heart disease. Quartile of dietary carotenoid intake (μg/day) with Q1 [0,1745], Q2 (1745,4963.5], Q3 (4963.5,11662.5], Q4 (11662.5,377178].

### Association between total and carotenoid subgroups intake and S-Klotho concentration

The total carotenoid intake was positively and significantly associated with S-Klotho concentration among the elderly population in the fully adjusted model, both as a continuous and categorical variable (**Table 2**). The fully adjusted model was adjusted with consideration of various factors, such as year, age, gender, ethnicity, marital status, education level, family income-to-poverty ratio, smoking, drinking status, eGFR, BMI, total energy intake, physical activity, medication use, SII, serum 25-hydroxyvitamin D, and self-reported chronic diseases. It was observed that individuals with the highest intake of total carotenoids had significantly increased S-Klotho levels compared to those with the lowest intake in the fully adjusted model (**Table 2**). Among the carotenoid subgroups, only intakes of α-carotene, β-carotene, and lutein with zeaxanthin were significantly correlated with increased S-Klotho concentration, while the relationship was not significant for β-cryptoxanthin and lycopene (**Table 3**).

**Table 2:** Multivariate linear analysis of the association between total carotenoid and serum Klotho level.

Total Carotenoid	Model I β (95%CI)	P	Model II β (95%CI)	P	Model III β (95%CI)	P
Continuous	10.87 (1.53,20.21)	0.02	9.60 (0.90,18.30)	0.03	8.40 (0.48, 16.31)	0.04
Quartiles						
Quartile 1	Reference		Reference		Reference	
Quartile 2	29.09 (1.63,56.55)	0.04	27.5 (0.28,54.71)	0.05	23.82 ( -4.08, 51.72)	0.09
Quartile 3	24.1 (-0.34,48.55)	0.05	23.55 (-0.75,47.85)	0.06	25.07 (0.70, 49.44)	0.04
Quartile 4	40.85 (8.57,73.12)	0.01	39.12 ( 6.90,71.35)	0.02	34.64 (0.34, 69.62)	0.04

Model I: non-adjusted model; Model II: adjusted for year and age; Model III: adjusted for year, age, gender, ethnicity, marital status, education level, family income-to-poverty ratio, smoking status, drinking status, estimated glomerular filtration rate, body mass index, total energy intake, physical activity, medication use, systemic immune-inflammation index, serum 25-hydroxyvitamin D, and self-reported chronic diseases.

**Table 3:** Multivariate linear analysis of associations between carotenoid subgroups and serum Klotho level.

Carotenoid subgroups (Continuous)	Model I β(95%CI)	P	Model II β(95%CI)	P	Model III β(95%CI)	P
α-arotene	13.77 (7.18,20.35)	<0.01	12.57 (5.67,19.47)	<0.01	10.80 (4.49, 17.11)	0.01
β-Carotene	15.58 (9.95,21.21)	<0.01	14.34 (8.59,20.08)	<0.01	11.58 (6.44, 16.71)	<0.01
β-Cryptoxanthin	8.50 (-0.92,17.93)	0.08	9.66 ( -0.40,19.73)	0.06	7.06 ( -2.16, 16.29)	0.13
Lycopene	-4.56 (-13.18,4.05)	0.29	-4.93 (-13.17, 3.31)	0.24	-2.92 ( -10.69, 4.86)	0.45
Lutein with zeaxanthin	15.44 (7.70,23.19)	<0.01	14.1 (6.63,21.58)	<0.01	10.33 (2.02, 18.64)	0.02

Model I: non-adjusted model; Model II: adjusted for year and age; Model III: adjusted for year, age, gender, ethnicity, marital status, education level, family income-to-poverty ratio, smoking status, drinking status, estimated glomerular filtration rate, body mass index, total energy intake, physical activity, medication use, systemic immune-inflammation index, serum 25-hydroxyvitamin D, and self-reported chronic diseases.

### Subgroup and sensitivity analysis

In stratified analyses, significant associations between α-carotene, β-carotene, lutein with zeaxanthin, and S-Klotho concentration were consistent among aged males, normal-weight individuals, and those with moderate physical activity (**Table 4**). For obese individuals, only β-cryptoxanthin intake was positively and significantly associated with S-Klotho concentration, while there was no significant relationship for α-carotene, lycopene, β-carotene, and lutein with zeaxanthin (**Table 4**). To further validate the robustness of our findings, we conducted sensitivity analyses that incorporated several macronutrients (carbohydrates, proteins, and sugars) into our multivariable linear regression models. The relationship between total carotenoids and S-Klotho concentrations did not change significantly. (**Table S2**).

**Table 4:** Stratified analysis of associations between carotenoid subgroups and serum Klotho level.

Carotenoid subgroups (continuous)	Model III β(95%CI)	P-value
Stratified by Gender		
Female		
α-Carotene	8.55( -15.54, 32.63)	0.48
β-Carotene	13.22( -3.83, 30.27)	0.13
β-Cryptoxanthin	4.58( -2.99, 12.16)	0.23
Lycopene	4.55( -8.25, 17.34)	0.48
Lutein with zeaxanthin	1.91( -12.85, 16.67)	0.80
Male		
α-Carotene	10.99( 7.71, 14.27)	<0.01
β-Carotene	11.09( 7.02, 15.16)	<0.01
β-Cryptoxanthin	22.25( -11.25, 55.76)	0.19
Lycopene	-9.09( -19.12, 0.94)	0.07
Lutein with zeaxanthin	14.42( 7.14, 21.69)	<0.01
Stratified by body mass index		
Normal weight		
α-Carotene	8.81( 3.90, 13.71)	<0.01
β-Carotene	11.5( 8.12, 14.88)	<0.01
β-Cryptoxanthin	-3.59( -28.91, 21.73)	0.78
Lycopene	-9.8( -29.36, 9.76)	0.32
Lutein with zeaxanthin	13.34( 6.36, 20.32)	<0.01
Overweight		
α-Carotene	-1.95( -24.05, 20.14)	0.86
β-Carotene	-6.73( -26.98, 13.52)	0.51
β-Cryptoxanthin	3.81( -2.30, 9.92)	0.22
Lycopene	7( -7.50, 21.50)	0.34
Lutein with zeaxanthin	-12.52( -33.08, 8.05)	0.23
Obesity		
α-Carotene	40.6( -11.84, 93.05)	0.13
β-Carotene	32.64( -1.35, 66.63)	0.06
β-Cryptoxanthin	33.31(6.25, 60.38)	0.02
Lycopene	-9.65( -24.89, 5.58)	0.21
Lutein with zeaxanthin	24.53( -2.84, 51.91)	0.08
Stratified by physical activity		
With moderate physical activity		
α-Carotene	9.64( 3.54, 15.74)	<0.01
β-Carotene	10.71( 5.20, 16.21)	<0.01
β-Cryptoxanthin	5.52( -1.89, 12.92)	0.14
Lycopene	-2.61( -12.83, 7.62)	0.61
Lutein with zeaxanthin	13.11( 5.27, 20.95)	<0.01
Without moderate physical activity		
α-Carotene	28.95( -15.81, 73.70)	0.20
β-Carotene	19.68( -3.67, 43.02)	0.10
β-Cryptoxanthin	21.21( -21.48, 63.90)	0.32
Lycopene	-5.87( -23.10, 11.37)	0.50
Lutein with zeaxanthin	-1.46( -24.53, 21.61)	0.90

Model III adjusted for year, age, gender, ethnicity, marital status, education level, family income-to-poverty ratio, smoking status, drinking status, estimated glomerular filtration rate, body mass index, total energy intake, physical activity, medication use, systemic immune-inflammation index, serum 25-hydroxyvitamin D, and self-reported chronic diseases. The strata variable was not considered when performing the stratification analysis.

## Discussion

This cross-sectional analysis investigated the correlation between the dietary total carotenoid intake (including five carotenoid subgroups) and the S-Klotho plasma level in aged adults. Despite the emerging evidence of a relationship between dietary pattern and Soluble Klotho (S-Klotho) levels (23, 24, 29–33), further evaluation is needed to assess the association between dietary carotenoid intake and S-Klotho levels among the elderly population. Therefore, we demonstrated that the total carotenoid intake was associated with the increased S-Klotho level, even after adjusting for potential confounding factors. The fact that the β-coefficient was shown to be significant whether total carotenoid intake was examined as a continuous or quartile variable suggests that this finding may have important clinical implications. The positive relationship between α-carotene, β-carotene, and lutein with zeaxanthin intake and the S-Klotho level was still evident. In subsequent analyses stratified by gender, BMI, and physical activity, the positive associations of α-carotene, β-carotene, and lutein with zeaxanthin were statistically significant among male aged participants as well as those with normal weight, and with moderate physical activity.

As life expectancy increases, the challenges of an ever-growing segment of the population facing aging and age-related illnesses continue to emerge. Elderly individuals are more susceptible to oxidative stress due to weakened endogenous antioxidant systems. As antioxidants, carotenoids are thought to help protecting the body from oxidative damage caused by free radicals and reactive oxygen species, which can be accumulated over time and contribute to age-related diseases. Numerous studies have found a correlation between the carotenoid intake and the reduced oxidative stress (20). As a result, the role of carotenoids in promoting healthy aging through countering oxidative stress is a topic that deserves further attention (21). In fact, carotenoids are a group of pigments that are produced exclusively by plants, algae, and certain bacteria. There are over 600 types of carotenoids that have been identified, but only around 50 of them are commonly found in the human diet. Among these, β-carotene, α-carotene, β-cryptoxanthin, and lycopene are the most extensively researched carotenoids due to their high prevalence in the diet and potential health benefits. In more detail, α-Carotene is a yellow-orange pigment that is present in similar food sources as β-carotene, including spinach, broccoli, pumpkin, mango, apricots, and citrus fruits such as oranges and tangerines. As for the β-Carotene, it is a red-orange pigment found in fruits and vegetables, particularly those with deep green, yellow, or orange hues. It is most abundantly found in sweet potatoes, carrots, pumpkin, spinach, kale, collard greens, and cabbage. Moreover, the β-Cryptoxanthin is an orange-red carotenoid that is found in numerous fruits and vegetables, particularly in deep green leafy vegetables and citrus fruits. Some of the richest sources of this caronete include kale, parsley, spinach, persimmons, winter squash, papaya, and tangerines. Finally, lycopene is a red pigment primarily found in tomatoes, watermelon, pink grapefruit, guava, and apricots, with tomatoes and tomato products being the richest dietary sources of this carotenoid. A meta-analysis, based on Liu et al., demonstrated that zeaxanthin and lutein therapy could reduce the risk of cataracts (34). Evidence suggests that individuals, who frequently eat carotenoid-rich foods, are less likely to suffer from age-related macular degeneration than those who rarely or never consume carotenoids (35). Numerous studies have showed that intakes of carotenoid subgroups, such as zeaxanthin and lutein, can improve visual performance, for example, photo stress recovery, contrast sensitivity, and glare tolerance (36, 37). Furthermore, Carotenoids may be able to reduce oxidative stress, conferring advantages of the ocular health and performance. Besides, carotenoid concentration is associated with better cognitive performance in both healthy and impaired individuals (38, 39). It was further demonstrated that a one-year supplementation of lutein, zeaxanthin, and meso-zeaxanthin had a beneficial effect on memory (40). Finally, epidemiological research has demonstrated that consuming a diet high in carotenoids could help reduce the likelihood of developing osteoporosis and heighten the bone mineral density (41, 42).

Long-term inflammation has a considerable impact on the decrease of Klotho in the serum (43). It has been observed that in middle-aged and older individuals, the plasma level of S-Klotho is inversely proportional to a pro-inflammatory dietary pattern as assessed by the Dietary Inflammatory Index (DII) (44). As for lower concentrations of S-Klotho, they have been associated with a variety of age-related medical conditions, including cancer, high blood pressure, and kidney disease (5, 45, 46). The aging process is often accompanied by a reduction in plasma S-Klotho levels, as evidenced by multiple studies (47–49). It is possible to reduce the inflammation by consuming a diet rich in anti-inflammatory or antioxidant components, such as fiber, nuts, fruits, and vegetables. We speculated that carotenoids might increase S-Klotho plasma levels by reducing oxidative stress and inflammation, particularly for α-carotene, β-carotene, lutein, and zeaxanthin. Subgroup analysis indicated that the beneficial effects of α-carotene, β-carotene, and lutein with zeaxanthin were more significant in male individuals who were older, had a normal weight, and exercised moderate physical activity. It had been suggested that the observed associations stratified by these factors might be attributed to a variety of potential causes. For example, research had indicated that there were sex-based differences in Klotho expression and function (50, 51), which could explain the greater association observed in males. Additionally, individuals with normal body weight tended to have better metabolic health and lower inflammation (52), which could play a role in the relationship. Furthermore, moderate physical activity had been linked to a range of health benefits, including improved insulin sensitivity, reduced inflammation, and enhanced antioxidant defense systems and immune function (53), which could also be contributing to the observed association. These findings required further prospective studies to validate and explain this relationship and their respective underlying mechanisms.

Using the large and nationally representative NHANES database, our findings reveal, for the first time, the presence of a positive correlation of the total carotenoid consumption – especially that of α-carotene, β-carotene, and lutein with zeaxanthin – with the higher level of S-Klotho in aging adults. The large sample size and various sensitivity analyses bolster the reliability of our results, which is our advantage. However, it is important to note that, due to the cross-sectional design of our study using the NHANES database, it is not possible to establish a causal relationship between the carotenoid intake and S-Klotho levels. Further prospective studies will be necessary to investigate this relationship and its underlying mechanisms. Additionally, we adopted two 24-hour dietary recalls to collect dietary data due to the lack of a validated Food Frequency Questionnaire (FFQ) in NHANES 2007-2016. The use of 24-hour dietary recalls for determining carotenoid intake necessarily incurred the risk of recall bias. Since the duration of supplementation with carotenoids was not available in the NHANES database, it may potentially impact the association between the carotenoid intake and the S-Klotho plasma levels. In addition, further studies should encompass more information regarding medications or other supplements that may alter the levels of carotenoids. An additional limitation was the absence of assessment of potential interactions between different variables in the stratified analyses. Despite taking into account certain potential confounding factors, there may be other unknown elements, such as antioxidants and oxidative stress indicators, that could affect our results. Further studies are required to assess the levels of antioxidants, oxidative stress markers, and inflammation response in order to uncover the underlying mechanism. Finally, careful consideration must be taken when attempting to generalize the findings of this study, which was conducted on a Western population, to other populations.

In conclusion, our findings showed a positive link between higher consumption of total carotenoids (especially α-carotene, β-carotene, and lutein with zeaxanthin) and increased levels of S-Klotho for older populations. These findings might provide a unique view on the part of dietary phytochemicals in age-related diseases and offer a comprehensive analysis into a potential intervention that could affect the aging process and thus encourage healthy longevity. Further research is needed to validate these findings and uncover the underlying mechanisms.

## Data availability statement

The original contributions presented in the study are included in the article/**Supplementary Material**. Further inquiries can be directed to the corresponding author.

## Ethics statement

The studies involving humans were approved by National Center for Health Statistics Ethics Review Board. The studies were conducted in accordance with the local legislation and institutional requirements. The participants provided their written informed consent to participate in this study. The manuscript presents research on animals that do not require ethical approval for their study.

## Author contributions

XH: Conceptualization, Investigation, Project administration, Resources, Supervision, Writing – original draft, Writing – review & editing. XY: Data curation, Formal Analysis, Methodology, Project administration, Validation, Writing – original draft. XC: Data curation, Formal Analysis, Methodology, Project administration, Validation, Visualization, Writing – original draft. XLC: Investigation, Supervision, Writing – review & editing.
