# Supplementary material for: Aging and antioxidants: the impact of dietary carotenoid intakes on soluble klotho levels in aged adults
Source: Front Endocrinol (Lausanne). 2023 Oct 26;14:1283722. doi: 10.3389/fendo.2023.1283722 (PMC10637857; doi:10.3389/fendo.2023.1283722)
Supplement: Supplementary file 1 [file Table_1.docx]

Supplementary material

**Table S1** **Characteristics of included population based on quartile of S-Klotho level in the NHANES (N =5,056)**

|  | | | **Quartile of S-Klotho level (pg/ml)** | | | |  |
| --- | --- | --- | --- | --- | --- | --- | --- |
| **Characteristic** | | **Overall**,  N = 5056 (100%) | **Q1**  N = 1264 (25%) | **Q2**  N = 1264 (25 %) | **Q3**  N = 1264(25%) | **Q4**  N = 1264 (25%) | **P Value** |
| Age (years) | | 67.50  (67.27,67.73) | 67.92  (67.52,68.32) | 67.54  (67.10,67.92) | 67.29  (66.83,67.76) | 67.23  (66.89,67.58) | 0.06 |
| Gender % | |  |  |  |  |  | <0.01 |
| Female | | 52.83 | 50.29 | 49.76 | 53.05 | 58.79 |  |
| Male | | 47.17 | 49.71 | 50.24 | 46.95 | 41.21 |  |
| Ethnicity % | |  |  |  |  |  | 0.02 |
| Non-Hispanic White | | 80.35 | 80.55 | 82.26 | 81.14 | 77.13 |  |
| Mexican American | 4.11 | | 4.07 | 3.88 | 4.23 | 4.25 |  |
| Non-Hispanic Black | 7.53 | | 7.2 | 6.43 | 6.6 | 10.14 |  |
| Other ethnicities | 8.02 | | 8.17 | 7.43 | 8.03 | 8.48 |  |
| Education % |  | |  |  |  |  | 0.75 |
| Grade or less | 16.57 | | 17.15 | 16.2 | 16.04 | 16.92 |  |
| High school | 22.94 | | 25.36 | 22.3 | 22.09 | 21.93 |  |
| Some college | 31 | | 29.98 | 30.8 | 32.77 | 30.38 |  |
| College or more | 29.5 | | 27.5 | 30.7 | 29.1 | 30.77 |  |
| Married status % | 66.73 | | 67.79 | 70.17 | 63.97 | 64.78 | 0.05 |
| Poverty income ratio | 3.20(3.09,3.30) | | 3.18(3.05,3.32) | 3.27(3.12,3.42) | 3.17(3.03,3.31) | 3.16(3.02,3.30) | 0.51 |
| Smoking status % |  | |  |  |  |  | 0.14 |
| Never | 47.05 | | 42.37 | 47.15 | 48.9 | 50.05 |  |
| Former | 40.61 | | 43.46 | 41.66 | 39.33 | 37.73 |  |
| Now | 12.34 | | 14.17 | 11.19 | 11.77 | 12.23 |  |
| Drinking status % |  | |  |  |  |  | <0.01 |
| Never | 12.66 | | 9.56 | 14.72 | 12.12 | 14.38 |  |
| Former | 22.25 | | 24.47 | 20.08 | 20.36 | 24.3 |  |
| Mild | 45.54 | | 42.89 | 45.23 | 46.95 | 47.24 |  |
| Moderate | 12.71 | | 13.93 | 13.06 | 13.4 | 10.23 |  |
| Heavy | 6.84 | | 9.15 | 6.91 | 7.18 | 3.85 |  |
| Body Mass Index (kg/m2) | 29.62(29.31,29.93) | | 29.92(29.45,30.38) | 29.28(28.77,29.79) | 29.71(29.09,30.32) | 29.58(28.91,30.24) | 0.33 |
| Energy intake (kcal/day) | 1899.36  (1867.60,1931.12) | | 1880.12  (1815.92,1944.32) | 1895.17  (1840.98,1949.36) | 1926.02  (1856.88,1995.17) | 1895.86  (1834.74,1956.98) | 0.8 |
| Physical activity (Yes) % | 70.66 | | 69.35 | 73.12 | 71.32 | 68.69 | 0.33 |
| Glomerular filtration rate (ml/min/1.73m^2^) | 75.94(75.12,76.77) | | 72.46(70.79,74.13) | 75.17(73.78,76.55) | 77.25(75.96,78.55) | 79.20(78.00,80.39) | < 0.01 |
| Serum 25-hydroxyvitamin D (nmol/L) | 78.31(76.58,80.05) | | 79.51(76.75,82.27) | 77.18(74.56,79.79) | 78.52(76.08,80.96) | 78.04(74.75,81.33) | 0.65 |
| Systemic immune-inflammation index | 553.67  (539.27,568.08) | | 603.83  (578.49,629.17) | 563.89  (539.81,587.97) | 540.44  (510.79,570.09) | 501.79  (485.58,518.00) | < 0.01 |
| Use of medication (Yes) % | 85.67 | | 89.05 | 85.15 | 83.59 | 84.84 | 0.04 |
| S-Klotho level (pg/ml) | 815.59  (802.59,828.60) | | 527.47  ( 520.73, 534.21) | 707.87  ( 705.16, 710.59) | 864.96  (860.90, 869.01) | 1197.44  (1172.11,1222.78) | < 0.01 |
| Total Carotenoid (μg/day) | 9775.25  (8971.30,10579.21) | | 9370.99  (8347.12,10394.87) | 9564.16  (8694.72,10433.60) | 9206.01  (8264.78,10147.24) | 11078.83  (8825.70,13331.96) | 0.25 |
| α-Carotene (μg/day) | 477.30  (389.28,565.32) | | 409.88  (311.19, 508.58) | 450.66  (383.69, 517.64) | 368.29  (313.89, 422.68) | 700.65  (341.42,1059.89) | 0.03 |
| β-Carotene (μg/day) | 2548.41  (2255.66,2841.16) | | 2133.65  (1823.28,2444.01) | 2508.31  (2163.95,2852.67) | 2177.49  (1962.20,2392.77) | 3455.79  (2306.29,4605.29) | 0.08 |
| β-Cryptoxanthin (μg/day) | 88.40  (80.42,96.39) | | 73.24  (63.55, 82.93) | 90.18  (75.65,104.72) | 92.81(77.14,108.48) | 98.24  (78.32,118.16) | 0.05 |
| Lycopene (μg/day) | 4943.17  (4524.48,5361.86) | | 5291.47  (4559.08,6023.85) | 4839.87  (4223.34,5456.39) | 4953.76  (4141.43,5766.09) | 4663.80  (4001.75,5325.84) | 0.64 |
| Lutein with zeaxanthin (μg/day) | 1717.97  (1536.96,1898.97) | | 1462.75  (1236.47,1689.04) | 1675.13  (1451.93,1898.33) | 1613.67  (1378.15,1849.20) | 2160.35  (1522.73,2797.97) | 0.21 |
| Self-reported chronic diseases | | | | | | | |
| CHD % | 8.83 | | 10.48 | 7.94 | 9.01 | 7.82 | 0.25 |
| Cancer % | 23.64 | | 27.94 | 21.82 | 22.27 | 22.43 | 0.03 |
| CHF % | 5.67 | | 7.26 | 4.87 | 5.38 | 5.14 | 0.16 |
| Stroke % | 5.87 | | 6.16 | 5.71 | 5.53 | 6.09 | 0.93 |
| Heart attack % | 7.34 | | 8.52 | 5.94 | 7.68 | 7.22 | 0.23 |
| Hypertension % | 56.73 | | 58.84 | 53.21 | 54.58 | 60.7 | 0.05 |
| Diabetes mellitus % | 19.43 | | 23.14 | 18.13 | 16.87 | 19.62 | 0.06 |
| Continuous variables are described as means ± 95%CI, and categorical variables are presented as percentages. All estimates accounted for complex survey designs. Abbreviations: CHF, Congestive heart failure; CHD, Coronary heart disease. | | | | | | | |
| Quartile of S-Klotho level (pg/ml) with Q1[152.5,635.075], Q2 (635.075,781.45], Q3 (781.45,966.975], Q4 (966.975,3694.8]. | | | | | | | |

**Table S2.** Multivariate linear analysis of the association between total carotenoid and serum Klotho level.

| **Total Carotenoid** | **Model I**  **β(95%CI)** | **P** | **Model II**  **β(95%CI)** | **P** | **Model III**  **β(95%CI)** | **P** |
| --- | --- | --- | --- | --- | --- | --- |
| Continuous | 10.72(1.32,20.12) | 0.03 | 9.42(0.64,18.20) | 0.04 | 8.67(0.57, 16.77) | 0.04 |
| Quartiles |  |  |  |  |  |  |
| Quartile 1 | Reference |  | Reference |  | Reference |  |
| Quartile 2 | 28.58(1.00,56.16) | 0.04 | 25.99( -0.25,52.22) | 0.05 | 25.78( -7.13, 58.70) | 0.12 |
| Quartile 3 | 25.61(0.20,51.02) | 0.05 | 24.96( -0.40,50.31) | 0.05 | 28.27(0.64, 55.91) | 0.05 |
| Quartile 4 | 40.47(8.16,72.77) | 0.01 | 37.85( 5.57,70.12) | 0.02 | 37.49(0.79, 75.77) | 0.04 |

Model I: non-adjusted model; Model II: adjusted for year and age; Model III: adjusted for year, age, gender, ethnicity, marital status, education level, family income-to-poverty ratio, smoking status, drinking status, estimated glomerular filtration rate, body mass index, physical activity, medication use, dietary macronutrients (carbohydrate, fat, protein, sugars) and self-reported chronic diseases.
